# Bridging Financial Inclusion and Health Equity in LMICs: Evidence from a Half-Century of Bibliometric Data

**DOI:** 10.3390/ijerph23010096

**Published:** 2026-01-10

**Authors:** Hasan Mhd Nazha, Masah Alomari, Mhd Ayham Darwich

**Affiliations:** 1 Faculty of Mechanical Engineering, Institute of Materials, Technologies and Mechanics, Otto Von Guericke University Magdeburg, Universitätsplatz 2, 39106 Magdeburg, Germany; 2 Faculty of Hospitals Management, Al-Andalus University for Medical Sciences, Qadmus, Tartus P.O. Box 101, Syria; dr.masah.alomari@au.edu.sy; 3 Faculty of Biomedical Engineering, Al-Andalus University for Medical Sciences, Qadmus, Tartus P.O. Box 101, Syria; a.darwich@au.edu.sy; 4 Faculty of Technical Engineering, Department of Industrial Automation, Tartous University, Tartus P.O. Box 2147, Syria

**Keywords:** financial inclusion, health equity, sustainable development goals, bibliometric analysis, health disparities

## Abstract

**Highlights:**

**Public health relevance—How does this work relate to a public health issue?**

- This research addresses the critical, yet understudied, link between financial inclusion and health equity, directly supporting the objectives of SDG 3 (Good Health and Well-being) and SDG 10 (Reduced Inequalities).
- It highlights how limited financial inclusion exacerbates health inequalities, particularly in low- and middle-income countries (LMICs), by restricting access to necessary care and increasing the burden of out-of-pocket healthcare spending.

**Public health significance—Why is this work of significance to public health?**

- This is the first bibliometric analysis of this scope to examine eighty years of research at the intersection of financial inclusion and health equity, revealing a major gap where fewer than 0.3% of the extant literature has explored this area.
- The study establishes a foundational body of evidence and proposes a research agenda to guide future interdisciplinary work on utilizing financial instruments to promote equitable health outcomes.

**Public health implications—What are the key implications or messages for practitioners, policy makers and/or researchers in public health?**

- Policymakers and health professionals should integrate financial inclusion strategies—such as digital finance, microinsurance, and mobile money—into broader health equity frameworks.
- Researchers are encouraged to adopt interdisciplinary approaches and develop common metrics that correlate financial inclusion indicators directly with health equity outcomes.

**Abstract:**

Health equity and financial inclusion (FI) are at the core of the Sustainable Development Goals, yet their intersection remains critically under-studied. This bibliometric study maps this emergent and fragmented field by analyzing 24,140 publications from Scopus, PubMed, Web of Science, and Lens.org over five decades. Employing co-citation and co-word analysis via VOSviewer, chart research trends, governance frameworks, and policy linkages were systematically presented. The analysis reveals that less than 0.3% of the identified literature explicitly bridges financial inclusion with health outcomes, and direct investigations into health equity are virtually absent. Despite recent growth, fundamental gaps persist, including a lack of empirical studies on digital financial tools in low- and middle-income countries (LMIC) health contexts and insufficient focus on disadvantaged populations. As the first comprehensive empirical mapping of this nexus, this study underscores the urgency for scholarly and policy action to strategically leverage financial instruments for equitable healthcare access. The findings provide a foundational map and a structured agenda to consolidate this nascent field.

## 1. Introduction

Health equity (HE) defined as “the absence of avoidable or remediable differences in health among groups of people” remains one of the fundamental pillars of the Sustainable Development Goals (SDGs) [1]. Yet, entrenched inequalities, driven by healthcare access and financial exclusion underpinned by structural injustice, continue to undermine this vision [2,3]. Despite considerable progress in healthcare delivery, disadvantaged groups remain disproportionately included among those whose service needs, cost unaffordability, and adverse social determinants of health are low priorities [4,5].

Greater access to cheaper financial services—e.g., savings, credit, and insurance—is becoming more widely touted as a way to narrow these disparities [6,7]. By enabling coping with financial risk and out-of-pocket cost of health, financial inclusion can close the health equity-economic resilience gap [8]. FI is recognized as a catalyst for at least seven SDGs, including SDG 1 (No Poverty), SDG 3 (Good Health and Well-being), SDG 5 (Gender Equality), and SDG 10 (Reduced Inequalities) [6,9], yet scholarly attention to its intersection with health equity (a core aspect of SDG 3) remains fragmented.

Evidence from sub-Saharan Africa highlights FI’s critical role in addressing healthcare inequities. Financial exclusion has exacerbated barriers to surgical care and pain relief [10], while Community-Based Health Insurance (CBHI) schemes in Ethiopia face undercoverage and limited effectiveness [11]. In Ghana, social stigma and economic constraints reduce access to mental healthcare [12]. These cases illustrate the ways in which robust financial systems—particularly where underpinned by digital technology—can secure poor health systems.

Gender disparities also contribute to these problems. Women in LMICs, disproportionately excluded from formal financial systems, have accumulated healthcare barriers [13]. While digital financial tools (e.g., mobile money) promise to improve women’s health and financial well-being, socio-cultural norms and infrastructure shortfalls persist [14].

While bibliometric reviews in global health have examined topics like digital health [15] and health finance [16], they have not examined financial inclusion as an enabler of health equity. This study is situated within two allied theoretical paradigms: Sen’s capability approach, oriented toward human agency and freedom from deprivation [17], and the WHO’s social determinants of health paradigm [18], where financial and health inequities are structurally interrelated. These paradigms underpin the analysis of bibliometric trends, particularly gaps where research on financial inclusion overlooks health equity.

To our knowledge, no prior bibliometric study has systematically mapped the intersection of financial inclusion and health equity across multiple major databases. Previous reviews focus either on digital finance or on health inequities, but not on the conceptual linkage between the two domains.

Consequently, this study aims to systematically map and analyze the scholarly landscape at the intersection of financial inclusion and health equity. The specific objectives are: (1) to characterize the research landscape and its evolution; (2) to uncover the intellectual structure and key thematic clusters; (3) to identify dominant, niche, and emerging topics; (4) to propose a structured agenda for future research. This study contributes to the literature by providing the first comprehensive bibliometric mapping of the financial inclusion–health equity nexus, thereby systematically quantifying and visualizing the profound research gap that exists. By moving beyond a presentation of articles, this analysis connects the identified structural and thematic silos directly to actionable policy needs and interdisciplinary research priorities, offering a foundational evidence map to guide future efforts in leveraging financial systems for equitable health outcomes in LMICs.

With co-word and co-citation analysis, the development of the field and the identification of dominant research clusters and a thematic framework (motor, niche, emerging/declining, and basic themes) were followed. The findings emphasize the revolutionary promise of FI in LMICs and propose co-designed digital health-finance solutions to close gaps.

The paper adheres to the following format: Section 2 describes the methodology, Section 3 presents results, Section 4 deals with implications, and Section 5 concludes.

## 2. Materials and Methods

### 2.1. Search Strategy and Data Collection

Data were retrieved from four multidisciplinary databases—Scopus, PubMed, Web of Science, and Lens.org—selected for their comprehensive coverage of social sciences, medicine, and public health [19,20]. To capture the full spectrum of literature touching on both domains, a broad search strategy was designed using Boolean operators. The search terms combined two conceptual blocks:

Block 1 (Financial Inclusion): (“financial inclusion” OR “financial access” OR “digital finance” OR “mobile money” OR “fintech” OR “microfinance” OR “microcredit”).

Block 2 (Health Equity/Outcomes): (“health equity” OR “health disparity” OR “healthcare access” OR “social determinants of health” OR “health outcome” OR “universal health coverage”).

The search was conducted on article titles, abstracts, and keywords, and was limited to English-language publications from January 1944 to December 2024.

This initial search, intended to capture the broad disciplinary landscape surrounding both concepts, yielded 53,775 records. After removing duplicates, the combined corpus for initial bibliometric performance analysis (publication trends, journal analysis, broad keyword mapping) consisted of N = 24,140 unique publications.

### 2.2. Screening and Refinement for Thematic Analysis

**Figure 1:**
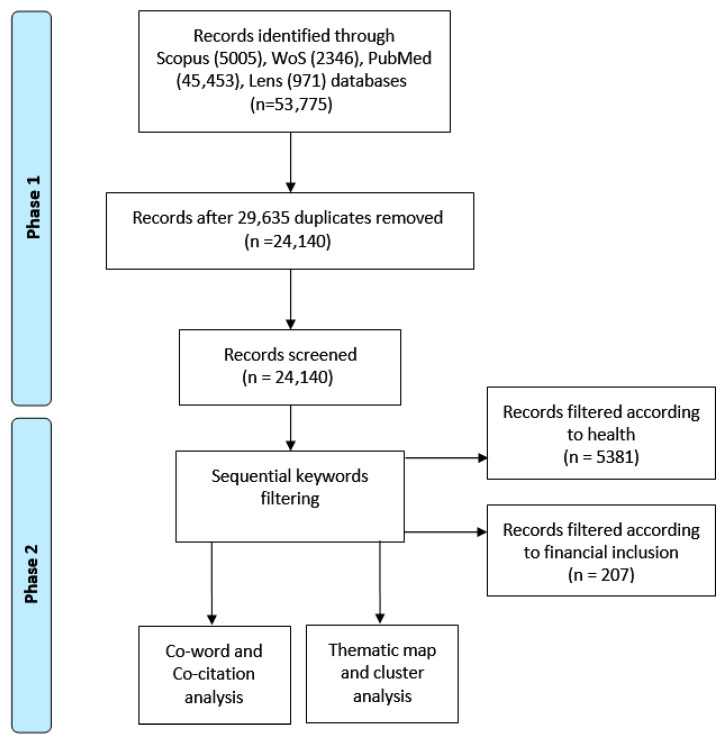
Workflow diagram of the bibliometric data collection, screening, and analysis process. Schematic of the two-phase bibliometric methodology. Phase 1 involved retrieving records from multiple databases to create a Broad Corpus for performance analysis. Phase 2 applied sequential keyword filtering to refine the dataset into a Thematic Corpus for science mapping.

To isolate literature explicitly examining the intersection of financial inclusion and health for thematic and network analysis, a two-stage screening process was applied to the 24,140 records, as detailed in Figure 1.

1. Title/Abstract Screening: Two reviewers independently screened titles and abstracts against the inclusion criterion: the study had to discuss a financial inclusion mechanism (e.g., savings, credit, insurance, digital payment) in relation to a health outcome, behavior, or access issue.
2. Full-Text Eligibility Assessment: The full texts of the 24,140 records were assessed. Studies were excluded if they (i) addressed financial access but without a clear link to health; (ii) discussed health disparities without a financial inclusion dimension; (iii) were non-empirical commentaries/editorials. From an initial pool of 24,140 articles, a sequential keyword filtration was applied. Filtering for ‘financial inclusion’ yielded 207 articles. A subsequent filter for ‘health’ within the initial subset yielded 5381 articles. To identify the most impactful works at the intersection for qualitative synthesis, the ten articles from this final set with the highest citation counts (n = 10) were isolated. This stringent filtering process—from 24,140 to a core intersection of 5381 articles—quantitatively underscores the paper’s central finding: the Financial Inclusion-Health Equity (FI-HE) nexus is profoundly under-studied, with most literature treating the domains in isolation.

### 2.3. Bibliometric Analysis Framework

The analysis followed a three-phase bibliometric framework, each designed to address a specific research objective.

**Phase 1: Performance Analysis (Addressing RQ1).** This phase characterized the field’s macroscopic profile using the broad initial corpus (N = 24,140). Publication trends, influential sources by applying Bradford’s Law, and subject area distributions were analyzed to map the broader disciplinary landscape.

**Phase 2: Science Mapping (Addressing RQ2 & RQ3).** To uncover the intellectual and conceptual structure of the core FI-HE nexus, science mapping on the refined corpus of 5381 articles identified through sequential keyword filtering were performed. The following steps were conducted using VOSviewer (version 1.6.20) [21]:

- **Co-word Analysis:** A network using both Author Keywords and KeyWords Plus as units of analysis was constructed. A minimum occurrence threshold was applied to ensure meaningful connections. The resulting network was visualized and thematically clustered using VOSviewer’s LinLog/modularity normalization method.
- **Thematic Map Analysis:** Based on the co-word network clusters, themes were categorized into four types—motor, niche, emerging, and basic—according to their centrality (relevance to the broader field) and density (internal development) metrics.

**Phase 3: Content Analysis.** A qualitative review of the most impactful publications, including the subset of 10 highly cited articles, was conducted to synthesize prevailing findings, proposed mechanisms, and critical research gaps. This synthesis directly informed the development of the discussion and the future research agenda.

## 3. Results

### 3.1. Research Landscape (Addressing RQ1)

This bibliometric study explores the evolving research terrain of financial inclusion as a pathway to health equity, spanning publications from 1944 to 2024. The multi-database corpus comprises 24,140 items after deduplication, drawn from 5005 Scopus, 45,453 PubMed, 2346 Web of Science, and 971 Lens.org records, with an average growth rate of 13.72% annually, indicative of growing academic interest in this multidisciplinary area. It is crucial to note that this broad corpus is used to analyze the field’s evolution and disciplinary footprint (RQ1). The subsequent conceptual structure analysis (RQ2/RQ3) is based on a refined subset of literature that explicitly links both concepts.

Although the global volume of publications related to finance, digitalization, and health is substantial, the intersection between financial inclusion and health equity remains extremely narrow. The magnitude of the initial dataset should therefore not be interpreted as maturity of the field but rather as an indication of disciplinary dispersion, since fewer than 0.3% of all retrieved papers examine financial inclusion in relation to health, and even fewer address health equity specifically.

**Figure 2:**
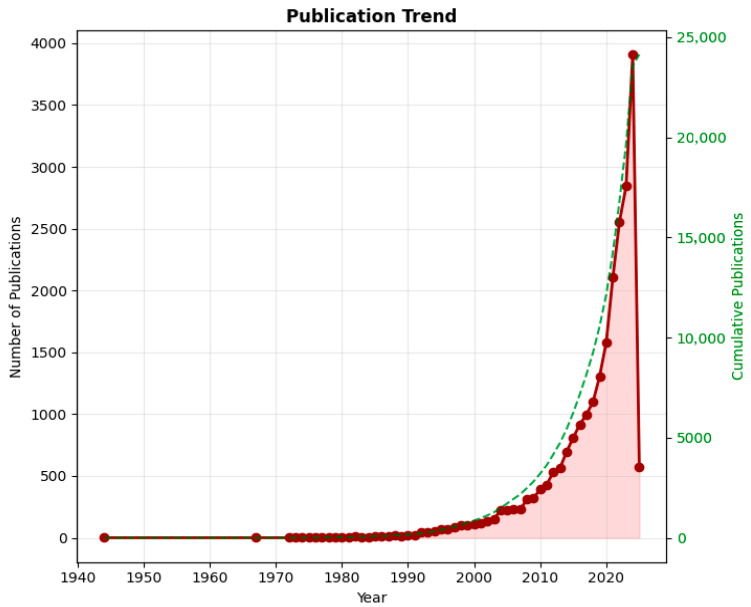
Annual publication trends (1944–2024) in financial inclusion and health equity research, showing the evolution of scholarly output and key growth periods.

The analysis reveals a notable surge in publications post-2000 (Figure 2). This growth trajectory aligns with major global policy milestones, including the establishment of the Millennium Development Goals in 2000 and their successor, the Sustainable Development Goals in 2015, which explicitly framed financial inclusion and health equity as interconnected development priorities [1,6,9]. Concurrently, the proliferation of digital financial technologies (e.g., mobile money, fintech) and the significant increase in donor funding for health systems research in LMICs seemed to provide both the subject matter and the resources that catalyzed scholarly interest at the intersection of finance and health.

**Figure 3:**
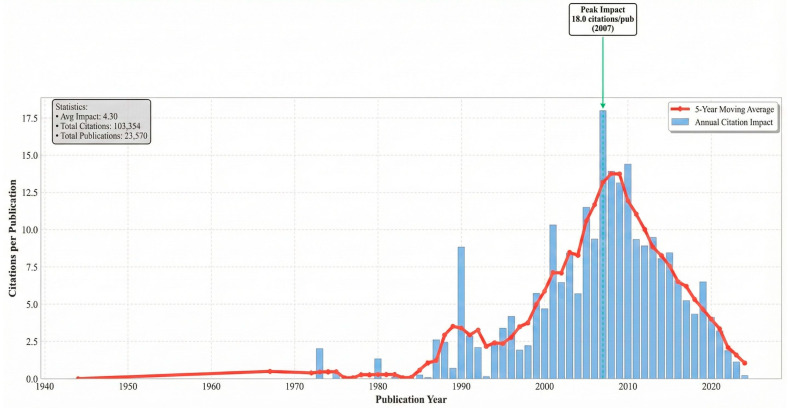
Temporal trends in citation impact for publications related to financial inclusion and health equity between 1944 and 2024. The figure illustrates the evolution of scholarly influence using citation-based indicators, including mean total citations per article and citation intensity over time.

Citation trends analysis was utilized from 1944 to 2024, with measures such as Mean Total Citations (TC) per Article, Mean TC per Year, and Citable Years utilized to quantify the scientific article’s impact and aging. Dataset size (N) offers safe analysis, and Mean TC per Year highlights citation impact over time trends. Citable Years puts article age into perspective to account for citation numbers, which reflect shifting patterns in scholarly impact. A sharp increase in the number of articles (N) across the years, particularly from the early 2000s and beyond, was noticed, peaking at 3800 articles in 2024. However, despite this productivity increase, Mean Total Citations (TC) per Article and Mean TC per Year both have a declining trend across the recent years, which indicates that while more articles are being created, their individual citation impact has declined as seen in Figure 3. This implies a possible weakening of citation impact with increasing numbers of publications. The decline in mean citations in the midst of publication increases suggests fragmentation of the field, where newer studies do not come together around shared paradigms.

Table 1 indicates the importance of citation-prone research in addressing global health and society issues, with most cited articles focusing on key issues such as climate change and health, clinical innovations, healthcare efficiency, and social determinants of health, such as mental health and poverty.

**Figure 4:**
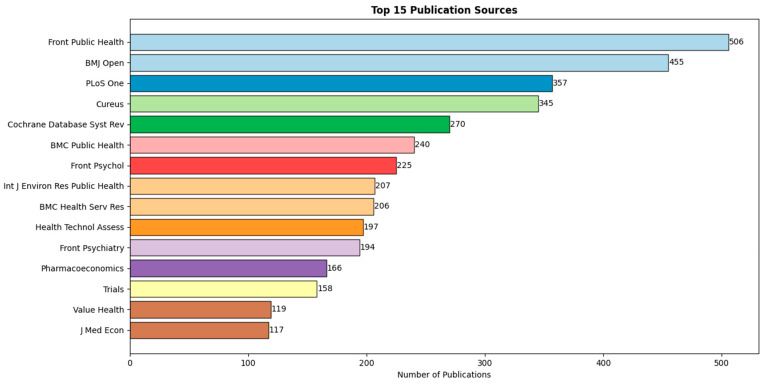
Most productive journals in the broad corpus. Journals like Frontiers in Public Health lead in volume, indicating where adjacent research is published, but not necessarily where integrative FI-HE studies appear.

The most influential resources in this field are presented in Figure 4. Leading journals driving the discourse include Frontiers in Public Health and BMJ Open (contributing 506 and 455 articles, respectively), followed by the PLoS One journal (357 articles).

This finding is further supported by Bradford’s law analysis, which categorizes journals into three zones based on their publication frequency. The top-ranked journals, such as Frontiers in Public Health, BMJ Open and PLoS ONE, have the highest number of publications, highlighting their prominence in the field, as outlined in Table 2.

A quantitative analysis of subject areas reveals the dominance of Public Health (59.24%) and Technology (18.69%), while Economics (9.28%) remain underrepresented, as shown in Table 3. The absence of a dedicated category for financial inclusion and health equity further emphasizes the need for this study, which bridges these disciplines to pioneer an interdisciplinary approach. By addressing this gap, the research provides actionable insights for policymakers and researchers, emphasizing the untapped potential of financial inclusion as a driver of health equity.

To study the nexus between financial inclusion and health in the literature, A three-step review was conducted. The articles that addressed the financial inclusion theme were initially reviewed. From an initial pool of 24,140 papers, a sum of 207 articles that addressed financial inclusion were screened by applying a title-based filter in the selected database.

The acquired evidence shows that those digital technologies, such as mobile banking, digital wallets, and fintech innovation, have significantly expanded financial access, particularly in developing economies [35]. These innovations empower marginalized communities like women and rural areas by providing them with access to credit, savings, and insurance [36,37,38].

Financial inclusion has strong links with broader socioeconomic benefits, such as reducing income inequality, enabling entrepreneurship, and spearheading progress toward the United Nations Sustainable Development Goals (SDGs), especially for low-income households and small businesses [7,39,40,41]. The overarching theme of the literature is the lack of access by females, with rural women facing disproportionate disadvantage. Closing this gap can consolidate economic empowerment, improve health outcomes, and reduce poverty [39,42,43,44].

Particularly, financial inclusion also enables access to medical care by decreasing out-of-pocket expenditures and providing opportunities for savings and insurance coverage during emergencies, particularly in low- and middle-income countries [45,46,47]. Financial literacy, inadequate infrastructure, and gaps in governance persist as perpetual challenges, nonetheless. Scholars bring into focus the necessity of particular policies to break through these hindrances [48,49,50]. Central Bank Digital Currencies (CBDCs) are also under investigation to facilitate financial inclusion with efficient, available digital payment facilities in under-banked regions [50,51,52].

To quantify the research gap, the refined corpus of 14 studies was analyzed. The ten most-cited articles within this set are presented in Table 4, demonstrating the early and limited evidence base. The concentration of publication years post-2019 confirms the field’s nascent state. A cross-check revealed zero overlap between these health-equity-focused papers and the FI-focused refined corpus, empirically confirming the conceptual siloing suggested by the network analysis.

This minimal number of publications indicates the early nature of research at the intersection of financial inclusion and health, leading to the need for additional study in this field. Despite these attempts, there is an astonishing dearth of research examining the direct nexus between financial inclusion and health equity, particularly in low- and middle-income countries (LMICs). This lacuna emphasizes the necessity of targeted research to explore ways through which financial inclusion can be leveraged in reducing health disparities and providing inclusive access to care. Collectively, the studies now reveal the revolutionary potential of financial inclusion towards better health outcomes, but they also prioritize understanding its unique contribution in achieving health equity.

Third, the articles that addressed health equity directly were deeply analyzed. Out of 24,140 documents, 52 papers were found to be associated with this particular topic when applying a title-based filter in the selected database (Table 5 shows the most recent). The suggested analysis reveals this gap empirically: less than 0.3% explicitly connect financial inclusion to health outcomes, and none specifically focus on health equity, underscoring a critical blind spot in the literature. The actual study synthesizes these results, presenting an integrated perspective on the field and setting the stage for strategic interventions to leverage financial inclusion to improve equitable health outcomes. By addressing these gaps, future research can provide actionable information on how financial inclusion can be leveraged as a tool for the alleviation of health inequities, particularly among marginalized groups.

### 3.2. Conceptual Structure (Addressing RQ2 & RQ3)

A co-occurrence network analysis, thematic map analysis, and thematic evolution analysis were conducted using VOSviewer software for bibliometric network visualization. Co-occurrence analysis identified six thematic clusters. The most frequent terms were “financial inclusion” (236), “systematic review” (143), “social determinants of health” (122), “COVID-19” (108), “mental health” (91), and “public health” (80). From the above discussion, financial inclusion initiatives, specifically microfinance, have demonstrated vast potential in preventing poverty as well as ensuring access to health care and education in the third world, though issues like usurious interest rates and poor policies persist. Fin-tech innovations such as mobile money and blockchain are revolutionizing the provision of financial services and healthcare but necessitate further improved digital literacy and cybersecurity measures. Health equity remains hindered by disparities in access, especially to vulnerable populations, and telemedicine has emerged as a key delivery mechanism to carry care beyond the pandemic. The mental health problems created by the COVID-19 pandemic exposed deficits in support systems for at-risk populations. Economic evaluation is centered on cost-containment healthcare policy, but funding and infrastructure deficits hamper progress. Central bank digital currencies and fintech platforms provide efficiency gains but face regulatory barriers. Cross-sector policy alignment among financial, health, and digital policies must be ensured to bridge systemic impediments and promote sustainable development goals.

Thematic mapping categorizes the identified keyword clusters into four distinct types based on their centrality (relevance across the field) and density (internal cohesion): motor themes (high centrality and density, driving the field), niche themes (high density but low centrality, specialized and developed), emerging/declining themes (low centrality and density, either rising or fading), and basic themes (high centrality but low density, foundational but underdeveloped).

**Figure 5:**
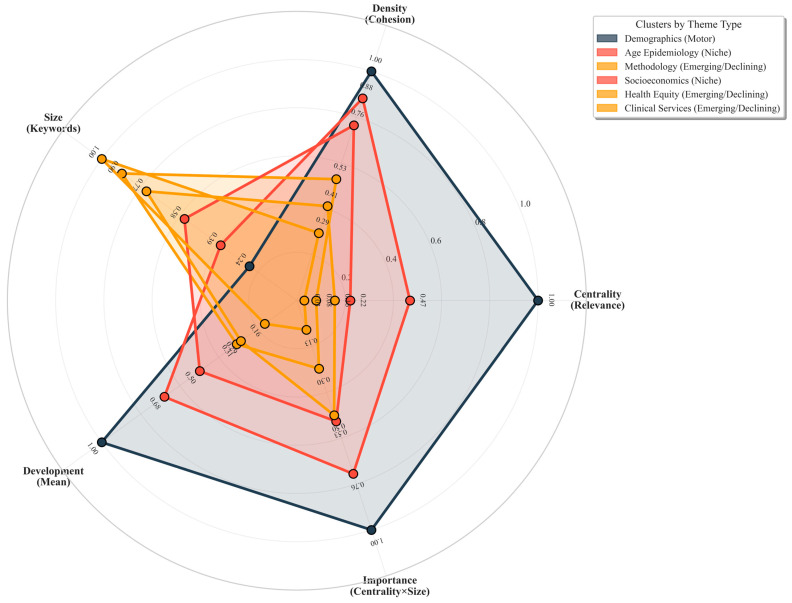
Radar-Type Thematic Map of Research Themes. This chart visualizes the four thematic categories—Motor, Niche, Emerging/Declining, and Basic—based on centrality (axis representing relevance to the broader field) and density (axis representing internal development). The position of each theme on the radar illustrates its role within the research landscape. Health equity themes appear in the outer, lower-density quadrant (Emerging/Declining), indicating their peripheral and underdeveloped status relative to core financial inclusion topics.

As illustrated in the radar-type thematic map (Figure 5), health equity-related terms (e.g., “health equity,” “social determinants of health”) occupy the emerging/declining quadrant with low density and centrality, visually confirming their peripheral position within the current scholarly landscape. In contrast, themes such as “financial inclusion” and “digital finance” exhibit higher centrality but are distributed across niche and basic quadrants, indicating they are recognized but not deeply integrated with health outcomes. This structural visualization underscores the fragmented and nascent state of research explicitly linking financial inclusion to health equity.

Table 6 summarizes six thematic clusters derived from the bibliometric analysis of 24,140 Scopus publications using three network metrics: (i) Betweenness Centrality identifies keywords that bridge disconnected themes, with the highest values observed in Cluster 1: Foundational Health Demographics (human = 1000.00, female = 960.05), indicating basic demographic identifiers serve as primary interdisciplinary gateways in health sciences research [67,68]. Notably, poverty demonstrates moderate bridging capacity but is distributed across clusters rather than concentrated, suggesting it connects financial and health themes in a fragmented manner. (ii) PageRank measures influence based on keyword co-occurrences [69]; dominant anchor themes like human (0.030), female (0.0289), and aged (0.0125) demonstrate high influence, with demographic terms rather than financial or equity concepts emerging as the most influential nodes in the network. (iii) Centrality/Density balances cross-theme relevance against thematic specialization [70]. The performed analysis reveals a critical structural pattern: Cluster 1 shows maximum connectivity (0.8235) but represents only core demographic concepts, while clusters containing substantive themes like Cluster 4: Socioeconomic Health Determinants (0.1825) and Cluster 5: Health Equity & Chronic Conditions (0.1295) show only moderate to low interdisciplinary bridging capacity.

Notably, financial inclusion concepts appear primarily in Cluster 4 with low centrality density values (0.041–0.085), indicating niche positioning with minimal integration into broader health equity discourse [43]. Poverty appears across multiple clusters but shows only moderate bridging capacity (Betweenness Centrality up to 175.03), suggesting fragmented rather than integrated treatment in the literature.

Despite the volume of clinical health services research (Cluster 6: 3065 keywords) and methodological studies (Cluster 3: 1338 keywords), few studies explore digital finance as a mechanism to reduce health disparities in low-income populations. The co-occurrence network analysis (5019 nodes, 636,427 edges) demonstrates that while socioeconomic determinants (Cluster 4) and health equity (Cluster 5) form distinct clusters, their moderate centrality metrics (0.1825 and 0.1295, respectively) indicate limited structural integration between financial access and health equity outcomes.

This research gap aligns with global priorities like WHO’s focus on economic tools for health equity and SDG 3.8 (universal health coverage), underscoring the need for interdisciplinary solutions that strengthen the financial inclusion–health equity nexus [71]. The disconnect persists despite evidence that socioeconomic factors moderately bridge financial and health domains, while core demographic concepts unexpectedly serve as the primary interdisciplinary connectors in health research literature.

**Figure 6:**
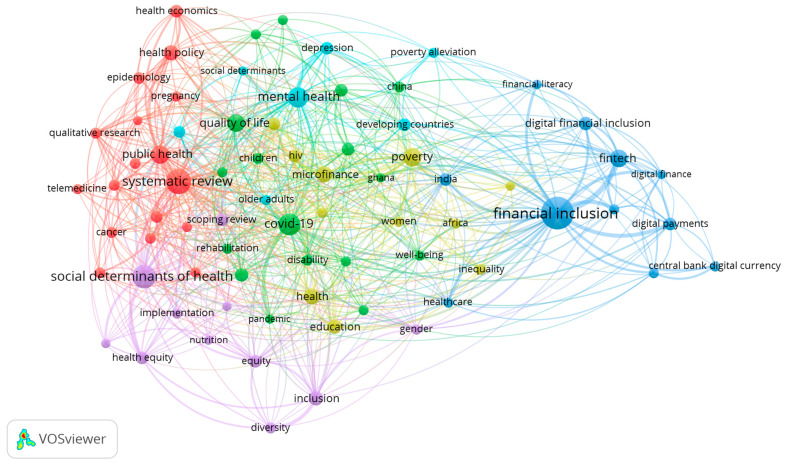
Co-occurrence network of keywords in financial inclusion and health equity research. The visualization maps the conceptual structure and thematic interconnections within the literature. Node size corresponds to keyword frequency, line thickness indicates association strength, and color denotes thematic clusters.

A co-occurrence network analysis was performed to reveal the underlying conceptual landscape of the research domain. The visualization (Figure 6) presents a structured map of how major themes cluster and interact. Larger nodes correspond to keywords that appear more frequently across the literature, while the thickness and intensity of the connecting lines reflect the strength of their co-occurrence. Together, these elements highlight the central hubs of discourse and the robustness of the relationships linking financial inclusion, health-related themes, and broader socioeconomic determinants.

Interpreting the clusters through the dual lenses of Sen’s Capability Approach and the WHO’s Social Determinants of Health (SDH) framework clarifies how financial inclusion shapes both individual capabilities and structural determinants of health. This theoretical triangulation strengthens the explanatory power of the bibliometric findings. Specifically, it illuminates how financial inclusion functions both as an enhancer of individual agency and a modifier of structural conditions. For instance, Cluster 4 (Socioeconomic Determinants) directly corresponds to the ‘social and economic context’ layer of the SDH model, while the mechanisms within it (e.g., health insurance) represent tangible instruments that expand individuals’ ‘capability sets’ to achieve good health, per Sen’s approach.

## 4. Discussion

### 4.1. Methodological Considerations

Several methodological considerations should be noted when interpreting these findings. First, bibliometric mapping reveals the structure and volume of research but does not assess the quality or contextual applicability of individual studies [72,73]. Second, while the suggested multi-database approach mitigates coverage bias, the inherent fragmentation of this nascent field means relevant studies may be indexed under keywords not captured by the suggested search strategy. Third, the focus on English-language, peer-reviewed literature may omit impactful policy reports, governmental documents, or locally generated innovations documented in grey literature or other languages, particularly from LMIC contexts where relevant interventions are being implemented [48]. This may limit the geographic and contextual diversity of the evidence base captured.

### 4.2. Key Findings and Interpretation

The analysis reveals significant intellectual fragmentation, characterized by three distinct and disconnected research clusters. Microfinance scholarship is pervasive yet seldom engages with health equity frameworks, despite their shared focus on vulnerability reduction [74]. Similarly, digital finance literature explores advanced technologies such as blockchain without systematically considering their potential to advance health equity [7]—a notable omission given documented secondary health benefits from innovations like M-Pesa in Kenya [75]. Parallel health equity studies continue to marginalize financial inclusion [5,76], notwithstanding increasing evidence that financial barriers constitute one of the most intractable social determinants of health. This fragmentation is reflected in low cluster density scores indicative of weak conceptual integration, a pattern consistent with pre-paradigmatic fields in early stages of consolidation [77].

The co-word and thematic analyses further demonstrate that while “financial inclusion” and “social determinants of health” appear within the network, their co-occurrence is weak. Financial inclusion concepts appear primarily in Cluster 4 (Socioeconomic Determinants) with low centrality and density values (Table 6), indicating niche positioning with minimal integration into the broader health equity discourse found in Cluster 5. This structural siloing suggests that financial inclusion research remains predominantly techno-centric and focused on aggregate economic outcomes, often overlooking its role as a modifier of structural conditions that produce health inequities, particularly for vulnerable populations in LMICs.

This gap represents a significant missed opportunity for policy integration. The persistent disconnect suggests that financial inclusion strategies are not being systematically evaluated or leveraged within health equity frameworks, despite their shared goal of reducing vulnerability. For policymakers, this indicates a need to consciously bridge these domains—for example, by designing health-finance products that explicitly target the financial barriers faced by populations identified in health equity research [5,76].

### 4.3. The Equity Blind Spot

Most notable is the field’s collective blind spot regarding equity dimensions. Although financial inclusion has been widely researched as a tool for poverty reduction and digitalization [6,78], the results indicate that health equity is rarely addressed directly. Equity-associated terms such as “vulnerable populations,” “universal health coverage,” and “social determinants of health” appeared in fewer than 5% of co-occurrence clusters. This suggests that equity remains peripheral rather than central to financial inclusion research—a missed opportunity to connect with ongoing work on health financing and social protection systems [79,80]. The persistent, well-documented barriers to care for underserved populations—from cancer [81] and mental health [82] to disability [83] and rural sexual health [84]—are compounded by financial exclusion, a factor that requires greater analytical emphasis. While financial exclusion is known to exacerbate inequalities in surgical access [85] and chronic disease management [86], few studies examine financial inclusion explicitly as a structural determinant of health equity. This gap echoes Farmer et al.’s critique of global health research for overlooking economic determinants of inequity [87] and is especially glaring given COVID-19′s demonstration of how financial risk translates directly into health risk [88].

### 4.4. A Path Forward

Moving forward, focused attention is needed in three interrelated areas to bridge the critical gaps identified in this bibliometric analysis and translate scholarly insight into tangible health equity gains:

1. Interdisciplinary research should move beyond descriptive mapping to clarify the causal pathways through which specific financial instruments affect equity metrics. This requires adopting robust experimental and quasi-experimental methods from development economics [89] within equity-focused evaluation frameworks [90]. For example, studies could investigate how microinsurance uptake influences health-seeking behavior for chronic diseases among rural women or how digital savings platforms affect maternal health outcomes in urban informal settlements.
2. Research must examine how fintech innovations can be intentionally harnessed within equitable health financing architectures. This involves learning from integrated systems like Kenya’s mobile money infrastructure to design ‘health-smart’ financial products [90] and India’s Aadhaar-linked welfare and health schemes [91]. Research should also investigate how financial resources are strategically allocated within health systems to improve equity [92,93], ensuring that digital tools do not exacerbate existing disparities in access.
3. Policy-Relevant Research and Monitoring should develop integrated monitoring frameworks that explicitly link financial inclusion indicators—such as those from the Global Findex [9]—with health equity outcomes, building on established tools like WHO’s health inequality monitoring framework [94]. This requires embracing insights from economics on evaluating trade-offs and policy options in public health [95] to provide policymakers with actionable evidence on which financial inclusion levers most effectively reduce disparities in healthcare access, financial risk protection, and health status for disadvantaged populations in LMICs.

### 4.5. Emerging Research Avenues

Several underexplored yet promising directions emerge from the structural gaps identified in the performed analysis. First, the convergence of digital health and finance offers opportunities to apply insights from behavioral economics [96], but must also address foundational barriers in low-income settings, such as those hindering telemedicine [97]. Second, intersectional analysis could deepen understanding of financial-health disparities, building on gendered exclusion research [98] and investigating how microfinance programs targeting women’s entrepreneurship translate into improved health agency. Third, crisis resilience research could adapt pandemic-era insights on financial safety nets [88] to advance long-term health equity, building on frameworks that view microfinance as a tool for climate change adaptation and health security [98]. Fourth, governance and policy-integration studies are needed to examine how fintech innovations can be harnessed within equitable health financing architectures, drawing lessons from integrated systems like India’s Aadhaar [91] to develop ‘health-smart’ financial products.

## 5. Conclusions

This paper provides the first comprehensive empirical mapping of the financial inclusion–health equity (FI–HE) nexus, revealing a profound research gap and underscoring the urgent need for both scholarly and policy engagement. The primary policy implication of this mapping is clear: financial and health sectors must move from operating in silos to coordinated action. Policymakers should use the identified gaps as a blueprint to foster ‘health-smart’ financial products and integrate financial inclusion metrics into health equity monitoring systems.

Three domain-specific research priorities emerge from the suggested analysis. First, digital finance scholars should pursue experimental evaluations of blockchain and other digital solutions for pro-poor health financing, building on models like Kenya’s M-Pesa but explicitly guided by equity objectives. Second, health economists should develop composite indices that align established financial inclusion metrics—such as the Global Findex—with health equity monitoring systems like the WHO’s Health Inequality Monitoring Framework, particularly for assessing programs such as maternal health microinsurance. Third, policymakers need implementation research on “health-smart” financial products—from disability-inclusive mobile wallets to gender-responsive credit mechanisms for healthcare loans—which directly address the exclusion patterns identified in the performed cluster analysis.

In the short term, strengthening conceptual integration will require advances in cross-disciplinary publishing and equity-oriented bibliometric reviews. Longer-term progress depends on the creation of standardized metrics linking financial inclusion data with health equity indicators, which can provide a consistent framework for evaluating how inclusion initiatives shape health outcomes. Alongside measurements, policymakers need robust evidence on the scalable application of financial tools that counteract structural health disadvantages.

While this study leverages a multi-database search across Scopus, PubMed, Web of Science, and Lens.org—covering 24,140 publications and offering a more comprehensive mapping than prior single-database reviews—certain limitations must be acknowledged. Bibliometric methods illustrate the structure of a field, not the quality of individual studies. Our focus on English-language, peer-reviewed literature may under-represent locally generated evidence and grey literature from LMICs, potentially omitting context-specific innovations at the finance–health interface. Future bibliometric work should incorporate multilingual sources and governmental or grassroots documentation to capture a more geographically diverse evidence base.

In closing, this inaugural map of the FI-HE nexus sounds a clarion call: integrating these currently siloed fields is not merely an academic exercise but a practical imperative for achieving the Sustainable Development Goals. Researchers must pursue embedded, interdisciplinary studies that trace the pathways from financial inclusion to health equity, while policymakers should foster innovation in ‘health-smart’ financial products, using the structural gaps identified here as a blueprint for action.

## Figures and Tables

**Table 1:** Most cited publications within the broad corpus (N = 24,140). This list reflects influential works in the adjacent fields of public health and economics, highlighting that highly cited research has not typically focused on the FI-HE nexus itself.

Ref. No.	Reference Title	Total Citation	TC Per Year
[22]	Costello, A.; Abbas, M.; Allen, A.; Ball, S.; Bell, S.; Bellamy, R.; Friel, S.; Groce, N.; Johnson, A.; Kett, M.; et al. Managing the health effects of climate change. *Lancet* 2009, 373, 1693–1733.	2095	26.18
[23]	Learmonth, I.D.; Young, C.; Rorabeck, C. The operation of the century: Total hip replacement. *Lancet* 2007, 370, 1508–1519.	1993	24.91
[24]	Zimlichman, E.; Henderson, D.; Tamir, O.; Franz, C.; Song, P.; Yamin, C.K.; Keohane, C.; Denham, C.R.; Bates, D.W. Health care-associated infections: A meta-analysis of costs and financial impact on the US health care system. *JAMA Intern. Med.* 2013, 173, 2039–2046.	1441	18.01
[25]	Zhang, W.; Nuki, G.; Moskowitz, R.W.; Abramson, S.; Altman, R.D.; Arden, N.K.; Bierma-Zeinstra, S.; Brandt, K.D.; Croft, P.; Doherty, M.; et al. OARSI recommendations for the management of hip and knee osteoarthritis. Part III: Changes in evidence following systematic cumulative update of research published through January 2009. *Osteoarthr. Cartil.* 2010, 18, 476–499.	1355	16.93
[26]	Donnelly, J.E.; Hillman, C.H.; Castelli, D.; Etnier, J.L.; Lee, S.; Tomporowski, P.; Lambourne, K.; Szabo-Reed, A.N. Physical activity, fitness, cognitive function, and academic achievement in children: A systematic review. *Med. Sci. Sports Exerc.* 2016, 48, 1197–1222.	1174	14.67
[27]	Babitsch, B.; Gohl, D.; von Lengerke, T. Re-revisiting Andersen’s Behavioral Model of Health Services Use: A systematic review of studies from 1998–2011. *GMS Psycho-Soc. Med.* 2012, 9, Doc11.	881	11.01
[28]	Lund, C.; Breen, A.; Flisher, A.J.; Kakuma, R.; Corrigall, J.; Joska, J.A.; Swartz, L.; Patel, V. Poverty and common mental disorders in low and middle income countries: A systematic review. *Soc. Sci. Med.* 2010, 71, 517–528.	851	10.63
[29]	Munro, S.A.; Lewin, S.A.; Smith, H.J.; Engel, M.E.; Fretheim, A.; Volmink, J. Patient adherence to tuberculosis treatment: A systematic review of qualitative research. *PLoS Med.* 2007, 4, e238.	740	9.25
[30]	Theorell, T.; Hammarström, A.; Aronsson, G.; Träskman Bendz, L.; Grape, T.; Hogstedt, C.; Marteinsdottir, I.; Skoog, I.; Hall, C. A systematic review including meta-analysis of work environment and depressive symptoms. *BMC Public Health* 2015, 15, 738.	714	8.92
[31]	Hout, M. Social and economic returns to college education in the United States. *Annu. Rev. Sociol.* 2012, 38, 379–400.	663	8.28
[32]	Bellis, M.A.; Hughes, K.; Ford, K.; Ramos Rodriguez, G.; Sethi, D.; Passmore, J. Life course health consequences and associated annual costs of adverse childhood experiences across Europe and North America: A systematic review and meta-analysis. *Lancet Public Health* 2019, 4, e517–e528.	642	8.02
[33]	Gomez, L.E.; Bernet, P. Diversity improves performance and outcomes. *J. Natl. Med. Assoc.* 2019, 111, 383–392.	619	7.73
[34]	Sabel, C.F.; Zeitlin, J. Learning from difference: The new architecture of experimentalist governance in the EU. *Eur. Law J.* 2008, 14, 271–327.	615	7.68

**Table 2:** Most contributing journals (n ≥ 200).

Journal	Publications	Ratio (%)
Front. Public Health	506	2.1
BMJ Open	455	1.88
PLoS One	357	1.48
Cureus	345	1.43
Cochrane Database Syst Rev	270	1.12
BMC Public Health	240	0.99
Front. Psychol.	225	0.93
Int. J. Environ Res. Public Health	207	0.86
BMC Health Serv. Res.	206	0.85

Source: Authors’ elaboration.

**Table 3:** Ratios of articles in the study database according to key subject areas.

Subject Area	Number Article	Ratio
Health/Medical	10,381	59.24%
Technology/Digital	3275	18.69%
Finance/Economics	1626	9.28%
Social Sciences	839	4.79%
Business/Management	482	2.75%
Education	444	2.53%
Policy/Governance	226	1.29%
Research Methodology	134	0.76%
Public Health	118	0.67%

**Table 4:** The most 10 cited papers addressed the intersection of financial inclusion and health.

Ref. No.	Paper Title	Total Citations	IF	Abstract of the Article
[53]	Gyasi, R.M.; Adam, A.M.; Phillips, D.R. Financial Inclusion, Health-Seeking Behavior, and Health Outcomes Among Older Adults in Ghana. *Res. Aging* 2019, 41, 794–820. https://doi.org/10.1177/0164027519846604	51	1.8	This study examines the associations between financial inclusion, health-seeking behavior, and health-related outcomes in older persons in Ghana. we estimated regression models of self-rated health (SRH), psychological distress (PD), and health-care use (HCU) on a variable representing compositional characteristics of financial inclusion.
[54]	Koomson, I., Abdul-Mumuni, A., & Abbam, A. 2021. Effect of financial inclusion on out-of-pocket health expenditure: Empirics from Ghana. *European Journal of Health Economics*, 22(9):14111425. https://doi.org/10.1007/s10198-021-01320-1	34	6.1	This study examines the link between financial inclusion and out-of-pocket health expenditure in Ghana. It finds that increased financial inclusion is associated with higher out-of-pocket health spending, especially among female-headed and urban households. The study suggests that scaling up financial inclusion can facilitate better health outcomes by enabling households to invest in health.
[55]	Alhanawi, M. K., Chirwa, G. C., Kamninga, T. M., & Manja, L. P. 2020. Effects of financial inclusion on access to emergency funds for healthcare in the Kingdom of Saudi Arabia. *Journal of Multidisciplinary Healthcare*,13:11571167. https://doi.org/10.2147/JMDH.S277357	15	4.6	This research investigates the effects of financial inclusion on access to emergency funds for healthcare in Saudi Arabia. It reveals that financially included individuals have a higher probability of accessing emergency funds and borrowing for medical purposes, particularly among low-income groups. The study underscores the need for policies that promote financial inclusion to enhance healthcare access.
[56]	Banerjee, R., Maruta, A. A., & Donato, R. 2023. Does higher financial inclusion lead to better health outcomes? Evidence from developing and transitional economies. *Economics of Transition and Institutional Change*, 31(2): 363–401. https://doi.org/10.1111/ecot.12341	9	1.7	This research analyzes the impact of financial inclusion on life expectancy and infant mortality in developing and transitional economies. It finds that financial inclusion improves health outcomes, particularly in societies with higher poverty and income inequality. The study emphasizes the role of financial inclusion in enhancing health capital and risk management.
[57]	Wirajing, M. A. K., Haruna, A., & Nchofoung, T. N. 2024. Financial inclusion and healthcare in Africa: Examining the moderating role of education. *Review of Development Economics*, 28(1): 97–127. https://doi.org/10.1111/rode.13043	3	3.2	This study investigates the effect of financial inclusion on healthcare in Africa, with a focus on the moderating role of education. It finds that financial inclusion enhances healthcare outcomes, particularly when combined with education and technology diffusion. The study suggests that financial literacy programs can improve healthcare in Africa.
[58]	Naveenan, R. V., Liew, C. Y., & Kijkasiwat, P. 2024. Nexus between financial inclusion, digital inclusion, and health outcomes: Evidence from developing economies. *Social Indicators Research*, 174(1): 367–408. https://doi.org/10.1007/s11205-024-03391-y	2	6.3	This research explores the nexus between financial inclusion, digital inclusion, and health outcomes in developing economies. It finds that digital inclusion moderates the impact of financial inclusion on health outcomes. The study suggests that policies promoting both financial and digital inclusion can enhance health indices in emerging markets.
[47]	Acheampong, A. O., & Tetteh, G. K. 2024. Does financial inclusion matter to population health? Insight from a global dataset. *Social Indicators Research*, 172(3): 1005–1040. https://doi.org/10.1007/s11205-024-03341-8	0	6.3	This global study investigates the relationship between financial inclusion and population health across 121 countries. It finds that financial inclusion improves health outcomes, especially in countries with lower income inequality and higher ICT penetration. The study highlights the importance of financial inclusion policies in promoting population health.
[59]	Ajide, F., Osinubi, T., Ojeyinka, T., & Kudaisi, B. V. 2024. The interactive effects of financial inclusion and women political empowerment on health outcomes in Africa. In *The Role of Female Leaders in Achieving the Sustainable Development Goals*: 136–159. https://doi.org/10.4018/979-8-3693-1834-8.ch008	0	N. A	This study examines the interactive effects of financial inclusion and women’s political empowerment on health outcomes in Africa. It finds that financial inclusion strengthens women’s political empowerment, leading to better health outcomes. The study highlights the role of financial inclusion in achieving Sustainable Development Goal 3 (SDG-3).
[60]	Nyamugira, A. B., Flessa, S., & Richter, A. 2024. Health insurance uptake, poverty and financial inclusion in the Democratic Republic of Congo. *Sustainable Development*, 32(4): 3293–3312. https://doi.org/10.1002/sd.2841	0	17.3	This research estimates the prevalence of health insurance coverage in the Democratic Republic of Congo and its association with financial inclusion. It finds that financial inclusion, proxied by bank account ownership, is strongly associated with higher health insurance coverage. The study recommends policies to improve financial inclusion to enhance health insurance uptake.
[61]	Saraswati, P. W. 2023. Corrigendum: Saving more lives on time: Strategic policy implementation and financial inclusion for safe abortion in Indonesia during COVID-19 and beyond. *Frontiers in Global Women’s Health*, 4: 1129026. https://doi.org/10.3389/fgwh.2023.1129026	0	3.7	This corrigendum corrects errors in a previous study on financial inclusion and safe abortion services in Indonesia. The original study highlights the importance of financial inclusion in providing safe abortion services, particularly through partnerships with the nonprofit private sector. The study underscores the role of financial inclusion in improving women’s health outcomes.

**Table 5:** The five most recent health equity papers in the studied sample.

Ref. No.	Paper Title	Total Citation	CiteScore	Abstract of the Article
[62]	Carmichael, A.E.; Lennon, N.H.; Qualters, J.R. Analysis of social determinants of health and individual factors found in health equity frameworks: Applications to injury research. *J. Saf. Res.* 2023, 87, 508–518. https://doi.org/10.1016/j.jsr.2023.10.001	1	6.9	Introduction: This research evaluated existing health equity frameworks as they relate to social determinants of health (SDOHs) and individual factors that may impact injury outcomes and identify gaps in coverage using the Healthy People (HP) 2030 key domains. Methods: The study used a list of health equity frameworks sourced from previous literature. SDOHs and individual factors from each framework were identified and categorized into the Healthy People 2030 domains. Five injury topic areas were used as examples for how SDOHs and individual factors can be compared to injury topic-specific health disparities to identify health equity frameworks to apply to injury research. Results: The study identified 59 SDOHs and individual factors from the list of 33 health equity frameworks
[63]	Kapalu, C.L.; Wilkes, C.D. Toward Promotion of Health Equity in Pediatric Disorders of Gut–Brain Interaction: A Call to Action. *Clin. Pract. Pediatr. Psychol.* 2023, 11, 449–464. https://doi.org/10.1037/cpp0000507	1	2.2	There has been increased attention to health equity, or the opportunity to obtain one’s health potential without disadvantage caused by discriminatory social systems, in recent years. The social determinants of health (SDoH), including economic stability, educational access and quality, healthcare access and quality, neighborhood and built environment, and social and community context, are the social, political, and systems-level factors that contribute to health inequities. In this commentary, the authors will review the ways in which structural and systemic racism impact health, discuss what is known about SDoH in pediatric DGBIs and propose a call to action for pediatric psychologists to promote health equity via research, clinical work, teaching, and advocacy.
[64]	Cook, N.E.; Kissinger-Knox, A.; Iverson, I.A.; Liu, B.C.; Gaudet, C.E.; Norman, M.A.; Iverson, G.L. Social Determinants of Health and Health Equity in the Diagnosis and Management of Pediatric Mild Traumatic Brain Injury: A Content Analysis of Research Underlying Clinical Guidelines. *J. Neurotrauma* 2023, 40, 1977–1989. https://doi.org/10.1089/neu.2023.0021	6	8.9	A content analysis of the literature underlying the Centers for Disease Control and Prevention (CDC) Guideline on the Diagnosis and Management of Mild Traumatic Brain Injury Among Children (i.e., the ‘‘Guideline’’) was conducted to determine the extent to which social determinants of health (SDoH) were examined or addressed. The systematic review forming the basis for the Guideline included 37 studies addressing diagnosis, prognosis, and treatment/rehabilitation. We examined those studies to identify SDoH domains derived from the U.S. Department of Health and Human Services’ Healthy People 2020 and 2030 websites.
[65]	Osae, S.P.; Chastain, D.B.; Young, H.N. Pharmacist role in addressing health disparities—Part 2: Strategies to move toward health equity. *JACCP J. Am. Coll. Clin. Pharm.* 2022, 5, 541–550. https://doi.org/10.1002/jac5.1594	10	2.6	Health disparities or extreme gaps of health across different groups of people result in poor outcomes for many Americans today. Pharmacists have an important role in addressing health disparities across many populations. Contextualizing root causes of gaps in health care and outcomes and identifying potential solutions to address those root causes are important initial steps clinical pharmacists can take to combat health disparities. PubMed and Web of Science were searched to identify articles published from inception until 9 May 2021.
[66]	Macintyre, A.K.; Shipton, D.; Sarica, S.; Scobie, G.; Craig, N.; McCartney, G. Assessing the effects of population-level political, economic and social exposures, interventions and policies on inclusive economy outcomes for health equity in high-income countries: A systematic review of reviews. *Syst. Rev.* 2024, 13, 1. https://doi.org/10.1186/s13643-023-02429-5	0	11.0	A fairer economy is increasingly recognised as crucial for tackling widening social, economic and health inequalities within society. However, which actions have been evaluated for their impact on inclusive economy outcomes is yet unknown. Objective: Identify the effects of political, economic and social exposures, interventions and policies on inclusive economy (IE) outcomes in high-income countries, by systematically reviewing the review-level evidence. We conducted a review of reviews; searching databases (May 2020) EconLit, Web of Science, Sociological Abstracts, ASSIA, International Bibliography of the Social Sciences, Public Health Database, Embase and MEDLINE; and registries PROSPERO, Campbell Collaboration and EPPI Centre (February 2021)

**Table 6:** Thematic Clusters: Key Metrics and Interpretations.

Cluster	Top 3 Keywords	Betweenness Centrality (Top Node)	PageRank (Top Node)	Centrality	Cluster Frequency	Interpretation
1. Foundational Health Demographics	Human, Female, Humans	1000.00 (human)	0.030 (human)	0.8235	6	Core demographic bridge concepts—Basic human identifiers serve as primary interdisciplinary connectors with maximum network centrality, linking diverse health research areas through common participant characteristics.
2. Age-Specific Health Epidemiology	Aged, Middle Aged, Child	386.27 (aged)	0.0125 (aged)	0.3862	22	Life stage health specialization—Focused research on age-specific health issues with strong bridging between pediatric, adult, and geriatric medicine, showing moderate interdisciplinary connectivity.
3. Health Research Methodology	Sensitivity Analysis, Awareness, Body Mass	24.76 (sensitivity analysis)	0.0010	0.0661	1338	Methodological and behavioral studies—Quantitative research techniques and health behavior investigations forming a large but specialized cluster with limited cross-theme connectivity.
4. Socioeconomic Health Determinants	Health Insurance, Education, Income	132.72 (health insurance)	0.0047 (health insurance)	0.1825	101	Financial and educational health influences—Research examining how economic resources and education impact health outcomes, showing moderate bridging capacity between financial and health domains.
5. Health Equity & Chronic Conditions	Health Equity, Obesity, Anxiety	42.37 (health equity)	0.0017 (health equity)	0.1295	487	Disparities in chronic disease burden—Studies addressing health inequities in the context of obesity, mental health, and other chronic conditions, with emerging interdisciplinary connections.
6. Clinical Health Services	Infection Risk, Children, Electronic Medical Record	7.33 (infection risk)	0.00036	0.0256	3065	Clinical operations and pediatric care—Largest cluster focused on healthcare delivery, infection control, and pediatric health services, highly specialized with minimal cross-theme bridging.

## Data Availability

The original data presented in this study are openly available in the Scopus, PubMed, Web of Science, and Lens.org databases. The specific search queries used to generate the dataset are available from the corresponding author upon reasonable request.
